# Detection of Incomplete Irreversible Electroporation (IRE) and Microwave Ablation (MWA) of Hepatocellular Carcinoma (HCC) Using Iodine Quantification in Dual Energy Computed Tomography (DECT)

**DOI:** 10.3390/diagnostics12040986

**Published:** 2022-04-14

**Authors:** Wolf Bäumler, Lukas Philipp Beyer, Lukas Lürken, Philipp Wiggermann, Christian Stroszczynski, Marco Dollinger, Andreas Schicho

**Affiliations:** 1 Department of Radiology, University Hospital Regensburg, Franz-Josef-Strauss-Allee 11, 93053 Regensburg, Germany; lukas.luerken@ukr.de (L.L.); christian.stros@ukr.de (C.S.); marco.dollinger@ukr.de (M.D.); andreas.schicho@ukr.de (A.S.); 2 Department of Diagnostic and Interventional Radiology, Ernst von Bergmann Hospital, Charlottenstraße 72, 14467 Potsdam, Germany; lukas.beyer@klinikumevb.de; 3 Department of Radiology and Nuclear Medicine, Hospital Braunschweig, Salzdahlumer Straße 90, 38126 Braunschweig, Germany; p.wiggermann@klinikum-braunschweig.de

**Keywords:** dual-energy computed tomography, irreversible electroporation, microwave ablation, hepatocellular carcinoma, residual tumor, iodine uptake

## Abstract

Early detection of local tumor progression (LTP) after irreversible electroporation (IRE) and microwave ablation (MWA) of hepatocellular carcinoma (HCC) remains challenging. The goal of this study was to identify cases with insufficient ablation and prevent HCC recurrencies by measuring iodine uptake using dual-energy computed tomography (DECT). In 54 HCC-patients, the volumetric iodine concentration (VIC) of the central and peripheral ablation area was evaluated by DECT within 24 h after IRE or MWA. Follow-up was performed with CT and/or MRI at 6 weeks, 3, 6, 9, and 12 months, respectively. In both groups, LTP was solely detected in the peripheral area (IRE: *n* = 4; MWA: *n* = 4) and LTP patients showed significantly higher VIC values in the peripheral zone than patients without LTP (IRE: * *p* = 0.0005; MWA: * *p* = 0.000). In IRE-LTP patients, no significant difference between the VIC values of non-ablated liver tissue and the peripheral zone was detected (*p* = 0.155). The peripheral zones of IRE patients without LTP (* *p* = 0.000) and MWA patients, irrespective of the presence of LTP (LTP: * *p* = 0.005; without LTP: * *p* = 0.000), showed significantly lower VIC values than non-ablated liver parenchyma. Higher BCLC tumor stages were indicative for LTP (* *p* = 0.008). The study suggests that elevated iodine uptake in the peripheral ablation zone could help identify LTP after IRE and MWA of HCC.

## 1. Introduction

Despite surgical resection being considered the most effective therapeutic option for hepatic malignancies, many patients cannot be treated by surgery because of several comorbidities or the advanced tumor stage [1]. For these patients, percutaneous ablation technology has evolved into an important treatment option. Irreversible electroporation especially (IRE), as a predominantly non-thermal ablative method, seems to offer advantages over thermal ablation techniques, such as microwave ablation (MWA). By causing cell death through the repeated application of high-voltage electrical impulses that generate irreversible damage to the membranes of tumor cells [2], IRE protects the architecture of adjacent structures such as vessels [1,3] and bile ducts [4], while thermal ablation techniques mostly entail the risk of damaging those contiguous structures [5,6]. Therefore, IRE in particular has acquired increased importance in recent years [7]. However, the use of percutaneous ablation techniques entails the remaining risk of primary incomplete ablation, as well as delayed local tumor progression from microscopically small residual tumors, as some ablation techniques, especially IRE, require the placement of several electrodes around the tumor, which is often located in close proximity to critical anatomical structures [8]. In order to reduce the risk of local tumor progression (LTP), the establishment of appropriate safety margins in ablations is of great concern. Both LTP, probably emerging from non-ablated microresidue, and incomplete ablation have to be identified as early as possible to evaluate retreatment or further therapeutic options. The current literature provides various numbers concerning post-ablative LTP of HCC, in some cases up to 20% [9]. Although magnetic resonance imaging represents an effective modality to detect hepatic tumors [10,11], it is often a challenge to differentiate between post-ablative tissue and LTP, as the post-ablative signal behavior of LTP may be inconsistent, especially in the case of HCC [12]. The evaluation of the post-ablative vascularization may represent an alternative method to identify LTP, especially in the peripheral part of the ablation area. Measuring the iodine concentration by using dual-energy computed tomography (DECT) is a method for evaluating the perfusion in abdominal computed tomography [13,14]. It can be applied to quantify the residual vascularization of post-ablative tissue after IRE and MWA of HCC, potentially helping to demask early LTP.

The aim of this study was to evaluate whether residual tumor tissue can be detected during the initial phase after IRE and MWA of HCC by analyzing the vascularization of the ablation area. Therefore, the iodine concentration of the ablation zone was quantified in DECT. Moreover, differences in vascularization in several parts of the ablation area were evaluated.

## 2. Materials and Methods

### 2.1. Study Design, Participant Selection and Patient Characteristics

The retrospective single-center observational study was approved by the local ethics committee (approval number 18-1027-104) and followed the guidelines outlined in the Declaration of Helsinki. The study was conducted in accordance with the relevant guidelines and regulations. To investigate the residual vascularization of the ablation area after IRE and MWA of HCC, the authors analyzed all available DECT images performed in the early phase after the interventions between February 2012 and November 2019. All patients underwent regular follow-up controls performed by contrast-enhanced computed tomography (CT), or even by additional magnetic resonance imaging (MRI). During the first year after IRE or MWA follow-up, CT imaging was regularly performed at 6 weeks, 3, 6, 9, and 12 months after the ablation. After the first postinterventional year follow-up, controls were conducted every 6 months. The inclusion criteria were as follows: (I) Histologically proven HCC. (II) HCC treated with IRE or MWA. (III) Examination by contrast enhanced DECT images of the entire liver within 24 h after the intervention and performance of follow-up imaging by CT at least 6 weeks, 3, 6, 9 and 12 months after the ablation. (IV) Cases of LTP had to be histologically proven. (V) Written informed consent was obtained from each patient for the acquisition of postinterventional contrast enhanced DECT images, follow-up MRI and/or CT, the ablation procedure, and the anonymous use of the data for scientific purposes.

Tumor stage was categorized according to the Barcelona Clinic Liver Cancer (BCLC) System [15]. In total, 54 patients (50 men and 4 women) fulfilled the set inclusion criteria. Among them, 27 were part of the patient group being treated with IRE and 27 were treated with MWA. The patient and disease characteristics are listed in Table 1.

### 2.2. Irreversible Electroporation (IRE)

All irreversible electroporations were performed percutaneously under CT-fluoroscopy guidance and full anesthesia using the NanoKnife system (AngioDynamics, Latham, New York, NY, USA). The operator placed two to six monopolar 18-gauge IRE probes parallel to each other in or around the target tumor, depending on size and position of the target lesion. The IRE parameters were as follows [16]: 70 pulses per cycle, pulse length of 90 µs; and electric field of 1500 V/cm needle distance with individual adaption following the manufacturer’s instructions (range: 1000–3000 V). A test pulse of 270 V was delivered before the delivery of 90 therapeutic pulses (range: 70–100 pulses) to confirm sufficient conductivity. If the current between two electrodes exceeded 48A (high-current condition), pulses were aborted to prevent heat induction.

### 2.3. Microwave Ablation (MWA)

All microwave ablations were performed under general anesthesia. After an initial 3-phase planning CT scan, the antenna placement was obtained manually with repeated verification of its position by using CT-fluoroscopy (CARE Vision, Somatom Sensation 16, Siemens Healthcare, Forchheim, Bavaria, Germany; CT parameters during fluoroscopy: tube voltage 120 kVp; effective tube current time product 30 mAs; slice collimation 16 mm × 0.75 mm) until the active feed point of the MWA antenna was in the tumor center. Depending on its relationship to the surrounding tissue and the tumor configuration, either the Acculis Microwave Tissue Ablation (MTA) System (AngioDynamics, Latham, New York, NY, USA) or the Emprint Ablation System (Medtronic, Minneapolis, Minnesota, MN, USA) was used.

### 2.4. Image Acquisition and Evaluation

Every patient underwent a standardized arterial and portal venous CT examination using a dual-source CT-system (SOMATOM Definition^®^ Flash, Siemens Healthcare, Forchheim, Bavaria, Germany) one day after the ablation. The DECT scan parameters were as follows: tube voltage, 80 kV and 140 kV; collimation 14 mm × 1.2 mm; rotation time 0.5 s; and pitch 0.6. To reduce the radiation dose, the manufacturer’s own automatic tube current modulation (CareDOSE 4D, Siemens Medical Solutions, Forchheim, Bavaria, Germany; reference current strength 550 mAs for tube A and 213 mAs for tube B) was applied. A virtual data set with 120 kV, 70% of which comprised the data from the 140 kV tube and 30% of the data from the 80 kV tube, was calculated from the raw data for the two tubes. The intravenous injection of 120 mL of the contrast agent (Accupaque 350, GE Healthcare Buchler, Braunschweig, Lower Saxony, Germany) was performed at a flow rate of 3–4 mL/s, followed by an injection of 40 mL of sodium chloride (NaCl). Acquisition in the arterial phase was performed using automatic bolus tracking (CARE Bolus, Siemens Healthcare, Forchheim, Bavaria, Germany) in the abdominal aorta at the level of the celiac artery with a threshold of 100 HU and a delay of 17 s. CT-imaging during the portal venous phase was obtained 75 s after injecting the contrast medium.

All DECT images were reconstructed as coherent axial slices. All data were transferred to a workstation compatible with the software syngo.via (Siemens Healthcare, Forchheim, Bavaria, Germany). The CT images were analyzed by two radiologists by consensus reading with then 4 years and 9 years of experience in abdominal imaging, respectively.

**Figure 1:**
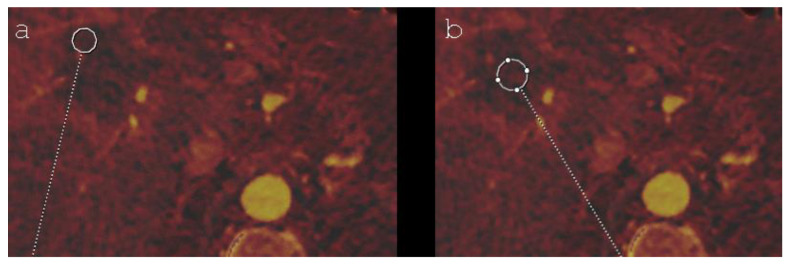
Axial DECT imaging (iodine mapping) shows an IRE ablation zone in segment V/VIII of a 72-year-old man, including the chosen regions of interest in its peripheral (**a**) and its central part (**b**).

The ablation zone was subdivided into a peripheral and a central part. The peripheral part was defined as the distance measured from the rim of the ablation zone that corresponds to 10% of its maximum diameter. The remaining part of the ablation area was defined as its center. By using a specific mode of the syngo.via software, the investigators evaluated the volumetric iodine concentration (VIC; measured in milligrams per millilitre) in the arterial phase CT-scan by choosing a region of interest in the central and the peripheral ablation zone (Figure 1). In order to have a reference value, the VIC of healthy liver parenchyma was also measured. In each region, five measurements were performed and the average was calculated for further statistical analysis. The measured volume of the central and peripheral ablation area was chosen as large as possible and a distance of more than 50% of its own diameter from adjacent tissue was required to minimize the volume averaging effect. For the healthy liver parenchyma, a diameter of 3.0 cm was defined for each region of interest. One year after the intervention, the results of the early-stage VIC measurements were compared with the imaging follow-up controls and, meanwhile, performed pathological results in case of LTP in order to evaluate their prognostic significance.

According to the International Working Group on Image-guided Tumor Ablation, the Interventional Oncology Sans Frontières Expert Panel, the Technology Assessment Committee of the Society of Interventional Radiology (SIR) and the Standard of Practice Committee of the Cardiovascular and Interventional Radiological Society of Europe (CIRSE), LTP describes the appearance of tumor foci after at least one study has documented adequate ablation and an absence of viable tissue in the target tumor, as well as in the surrounding ablation margin, regardless of when tumor foci were discovered either early or late in the course of imaging follow-up [17]. In clinical use, the term local tumor recurrence might be preferred. However, in this context, the term local tumor progression is more appropriate, as it must be assumed that local tumor recurrences are indeed the consequence of incomplete ablations, although no visible tumor tissue is verifiable in one or more postinterventional imaging modalities.

### 2.5. Statistical Analysis

All data are presented as frequency counts and percentages. To identify significant differences in the iodine uptake of the ablation area between patients with LTP and without LTP, a t-test for independent samples was used. A t-test for paired samples was utilized to evaluate differences in the iodine uptake of the different parts of the ablation area and the hepatic parenchyma of the IRE- and MWA-group, respectively. To identify factors for the prediction of LTP, a binary logistic regression model was used. As effect estimates, maximum-likelihood odds-ratio estimators and 95% confidence intervals are presented. A *p*-value of ≤ 0.05 was considered statistically significant. The analyzed variables were age, tumor stage, sex, tumor localization (segment I-IV vs. segment V-VIII) and the ablation method (IRE vs. MWA). All statistical analyses were performed with SPSS statistic (IBM SPSS Statistics, version 25, Armonk, New York, NY, USA).

## 3. Results

Both, the IRE and MWA group consisted of 27 patients, respectively. In each patient group, 25 men and 2 women were included. The mean age was 70.2 ± 9.5 years in IRE patients, and 67.6 ± 10.5 years in MWA patients. According to the BCLC System, tumor stage of the ablated HCC ranged from 0 to A3 in IRE patients and from 0 to A4 in MWA patients (Table 1). The mean diameter of the ablation area was 5.4 ± 0.9 cm in the IRE group and 5.4 ± 0.8 cm in the MWA group. In the IRE group, the ablated primary tumor was localized in each segment of the liver. In MWA patients, segments II–VIII were infiltrated by HCC (Table 1). All VIC values measured within the ablation zones were unequal to zero. The differences in the VIC in the various parts of the ablation zone and normal hepatic parenchyma all proved to be highly significant in each patient group (*p* = 0.000). The only exception was the difference between the mean VIC of the central and the peripheral ablation area of patients treated with MWA (*p* = 0.110; Table 2). All detected cases of LTP were localized in the peripheral ablation area. In the IRE group, 4 of 27 patients (14.8%) presented LTP of HCC, while in 4 of 27 patients (14.8%) treated with MWA, LTP was noticed. In the IRE group, only male patients suffered from LTP. In the MWA group, LTP could be proven both in men (*n* = 3) and women (*n* = 1). LTP was detected after 9 months (*n* = 3) and 12 months (*n* = 1) in the IRE group and after 6 months (*n* = 2), 9 months (*n* = 1) and 12 months (*n* = 1) in MWA patients. In both patient groups, a significant higher VIC was detected in the peripheral ablation area of patients with LTP after comparing them to patients without LTP (IRE group: * *p* = 0.005; MWA-group: * *p* = 0.000), Table 3. In IRE patients without LTP (* *p* = 0.000) and in MWA patients, irrespective of whether LTP of HCC had been detected (LTP: * *p* = 0.005; Absence of LTP: * *p* = 0.000), non-ablated liver parenchyma showed significantly higher VIC values than the periphery of the ablation area. In IRE patients suffering from LTP, no significant difference between the VIC values of non-ablated liver tissue and the peripheral ablation zone could be verified (*p* = 0.155). Table 4 summarizes the results. In the binary logistic regression model, tumor stage represented a significant influencing factor concerning the occurrence of LTP after IRE or MWA of HCC (*p* = 0.008). However, no significant correlation could be proven for the patients’ age (*p* = 0.451), sex (*p* = 0.379), the ablation method (IRE vs. MWA; *p* = 0.320) and tumor localization (segment I-IV vs. segment V-VIII; *p* = 0.524), Table 5. The mean period of follow-up was 28.2 months in the IRE group and 20.2 months in MWA patients.

## 4. Discussion

Representing a far less invasive method next to surgery, the importance of percutaneous ablation technology in the therapy of hepatic malignancies has increased during recent years. IRE, as a predominant non-thermal ablation technique, has especially attracted increasing attention [7]. Although many reports have been published covering the safety and efficacy of IRE and MWA [9,10,11,18], only little is known about the vascularization of the ablation zone and its potential significance concerning residual tumor tissue during the early phase after IRE and MWA of HCC. To our knowledge, this is the first study that systematically addresses the vascularization of the ablation zone by measuring the iodine uptake after an ablation therapy of HCC.

In four IRE patients (14.8%) and in four MWA patients (14.8%), LTP was detected during the follow-up control. In all eight patients, LTP was exclusively detected in the peripheral ablation zone, indicating an insufficient ablation. While “incomplete ablation” includes any residual visible tumor after ablation, “tumor progression” is defined as a newly detectable viable tumor after adequate ablation and the documented absence of visible tissue in the target tumor and in the surrounding ablation margin, most likely resembling residual untreated microscopic tumors [17]. Both incomplete ablation and LTP result in viable tumor tissue, potentially being identified at different points in time during follow-up control, based on the sensitivity of the utilized imaging method. In addition to a well-considered selection concerning follow-up imaging methods, the current trial emphasizes the importance of a sufficient safety margin in the ablation of liver tumors, as we currently cannot rely on microscopically validated R0 status after the ablation of hepatic tumors. These findings are consistent with other studies [19,20,21].

**Figure 2:**
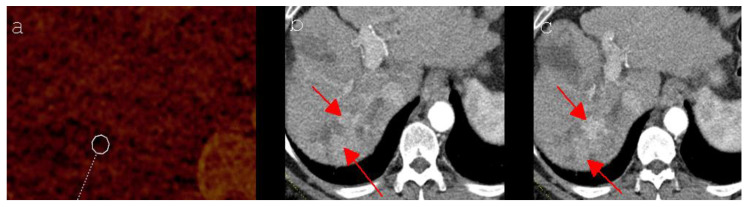
(**a**) Axial DECT imaging (iodine mapping) shows an ablation zone in segment VII of a 66-year-old man 24 h after MWA of a HCC, including the chosen regions of interest in its peripheral part. (**b**,**c**) Axial CT imaging (arterial phase) shows an arterial hypercasvular tumor mass (red arrows) in the peripheral part of the ablated area, which newly occurred at the 12 months follow-up control and turned out to be LTP of a HCC.

Although sharp demarcations between treated and untreated hepatic tissues could be detected in the histologic analysis of ablation areas [22,23,24], the exact visual assessment of ablation zones and potentially associated tumor tissue can be challenging using routinely applied imaging methods such as contrast-enhanced CT or MRI. In an animal study conducted by Guo et al. the post-imaging histologic analysis showed an overestimation of the visually identified IRE ablation zone, as the area of necrosis was distinctly smaller than supposed in the postinterventional MRI [25]. With the postinterventional biopsy followed by histologic analysis not being feasible in daily routine, additional imaging methods are of increasing interest. The assessment of iodine concentration seems to represent an appropriate available technique, which prevents the influence of hemorrhage and hyperdense coagulation by only reflecting the iodine-uptake ability of tissues [26]. Zhang et al., who assessed the therapeutic response after microwave ablation of HCC in a rabbit model, identified DECT-derived VIC as a potential imaging biomarker of tumor response, showing good reproducibility [26]. In the current study, all patients with LTP of HCC detected during imaging follow-up showed significant higher VIC values in the peripheral ablation zone of the 24 h postinterventional DECT, where the tumor was proven later on, than patients without LTP, irrespective of whether they had been treated with IRE or MWA (IRE patients: * *p* = 0.005; MWA patients: * *p* = 0.000). Consequently, the present study especially emphasizes the time-related advantages of DECT in the detection of residual tumor tissue. While LTP could be detected, at the earliest, 6 to 12 months after IRE or MWA by CT and/or MRI (Figure 2), DECT provided diagnostic clues within 24 h after intervention. This fact represents a crucial benefit compared to routinely applied imaging methods, as necessary retreatment can be initiated earlier, which may improve the patient’s outcome. Moreover, the measurement of the iodine uptake by DECT allows widespread utilization, as the dual-energy technique is currently available in many medical centers. Another important aspect is the fact that the performance of a postinterventional CT examination takes considerably less time than comparable methods such as MRI, merely requiring the appropriate software for the evaluation of iodine mapping. Furthermore, MRI follow-up controls might implicate the difficulty of inconsistent signal behavior of LTP [12], constituting an additional problem for the observer.

In IRE patients with LTP, no significant difference between the VIC values of non-ablated liver tissue and the peripheral ablation zone could be verified (*p* = 0.155), whereas the peripheral ablation zones of IRE patients without LTP (* *p* = 0.000) and MWA patients, irrespective of the presence of LTP (LTP: * *p* = 0.005; absence of LTP: * *p* = 0.000), showed significantly lower VIC values than non-ablated liver parenchyma. The almost equivalent iodine concentration measured in the peripheral ablation area and in non-ablated hepatic parenchyma, which was only detected in IRE patients with LTP, but not in MWA patients suffering from LTP, could possibly be based on a “fogging” phenomenon: the tumor itself, a transient effect called “reversible electroporation” [27], or a combination of both led to an increase in the VIC value, compensating the loss of iodine uptake caused by ablation. This theory might provide an explanation for the differences between patients of the IRE and MWA group, both suffering from LTP, and would be compatible with the findings of Padia et al., who assessed the post-IRE ablation zones of patients suffering from HCC by evaluating MR-imaging. The authors reported a temporary enhancement in the peripheral ablation zone, which had been detected on the first day after IRE but had disappeared during further follow-up. Padia et al. suggested that it might represent an area of “reversible electroporation” [27]. Based on the current trial, we are not able to prove this theory. Alternatively, a lack of change to the VIC in incomplete IRE needs to be taken into consideration.

Tumor stage, which was categorized according to the Barcelona Clinic Liver Cancer (BCLC) System [15], represented a significantly influencing factor concerning the occurrence of LTP (*p* = 0.008). These results coincide with the findings of Yu et al., who identified advanced tumor stage as a risk factor for LTP after local ablation therapy of HCC [28], and led to the assumption that an advanced tumor stage seems to represent an unfavorable prognosticator for the occurrence of LTP after IRE or MWA of HCC. To prove this hypothesis, further investigations are necessary.

To summarize, the mentioned aspects indicate that DECT-derived VIC could represent an auspicious imaging method helping to identify patients at risk for LTP after IRE and MWA of HCC at an early postinterventional stage, thus enabling early re-intervention and preventing tumor growth and spread.

The present study had several limitations: The first is its retrospective nature. The second limitation is the small sample size of the observed study population. Thirdly, although offering a long follow-up period using contrast-enhanced CT and/or MRI, the trial does not include further follow-up controls conducted by DECT. According to the national guidelines of HCC, the follow-up controls should be conducted every 3 months during the first year after successful local ablation therapy [29]. If LTP cannot be detected during the first postinterventional year, follow-up imaging should be conducted every 6 months for a total of 5 years [6,29]. Our cohort has a mean follow-up of 28.2 months in the IRE group and 20.2 months for MWA patients. Regarding the design of further investigations dealing with this topic, a longer follow-up should be considered. Additionally, the study groups consisted of a heterogenous patient population concerning sex and age. Nonetheless, to the best of our knowledge, this study provides the largest cohort addressing the highly relevant topic of identifying patients at risk for both incomplete IRE and MWA of HCC.

In order to initiate further investigations on this topic, a prospective study including a greater sample size and further follow-ups by DECT should be conducted. Moreover, the implementation of an iodine-uptake threshold referring to the iodine concentration measured in the non-ablated liver parenchyma could optimize the results and usability in everyday work. Furthermore, computer-assisted analysis could simplify the evaluation process and enable further insights using, e.g., techniques of artificial intelligence.

## 5. Conclusions

In conclusion, we suggest that elevated iodine uptake in the peripheral ablation zone identifies patients at risk of local tumor progression after both IRE and MWA of HCC. Furthermore, the trial illustrates that post ablation imaging and a sufficient safety margin during ablation are of utmost importance, as all cases of LTP were detected in the peripheral part of the ablation area. Moreover, the results of the study indicate that an advanced tumor stage seems to be a risk factor for the occurrence of LTP after IRE or MWA of HCC.

## Figures and Tables

**Table 1:** Patient and disease characteristics.

Characteristics	IRE	MWA
Number of patients	27	27
Age (years)		
Mean ± SD	70.2 ± 9.5	67.6 ± 10.5
Range	52−89	52−85
Sex, *n* (%)		
Male	25 (92.6)	25 (92.6)
Female	2 (7.4)	2 (7.4)
Diameter of the ablation area (cm)		
Mean ± SD	5.4 ± 0.9	5.4 ± 0.8
Tumor localization, *n* (%)		
Segment I	2 (7.4)	0 (0.0)
Segment II	1 (3.7)	3 (11.1)
Segment III	3 (11.1)	1 (3.7)
Segment IVa	3 (11.1)	1 (3.7)
Segment IVb	1 (3.7)	1 (3.7)
Segment V	7 (25.9)	7 (25.9)
Segment VI	4 (14.9)	4 (14.9)
Segment VII	1 (3.7)	3 (11.1)
Segment VIII	5 (18.5)	7 (25.9)
Tumor stage (BCLC System), *n* (%)		
0	2 (7.4)	2 (7.4)
A1	10 (37.0)	9 (33.3)
A2	8 (29.6)	6 (22.3)
A3	7 (26.0)	9 (33.3)
A4	0 (0)	1 (3.7)
B	0 (0)	0 (0)
C	0 (0)	0 (0)
D	0 (0)	0 (0)

IRE, Percutaneous irreversible electroporation; MWA, Microwave ablation; SD, Standard deviation; BCLC, Barcelona Clinic Liver Cancer.

**Table 2:** T-test for paired samples of the VIC after IRE and MWA in each patient group.

	IRE		MWA	
	*p*-Value	95% Confidence Interval	*p*-Value	95% Confidence Interval
Pair 1: Mean VIC (central ablation zone)–Mean VIC (peripheral ablation zone)	0.000	−0.85– (−0.44)	0.110	−0.28–0.03
Pair 2: Mean VIC (non-ablated liver parenchyma)–Mean VIC (central ablation zone)	0.000	0.96–1.31	0.000	0.44–0.94
Pair 3: Mean VIC (non-ablated liver parenchyma)–Mean VIC (peripheral ablation zone)	0.001	0.23–0.76	0.000	0.28–0.84

VIC, Volumetric iodine concentration; IRE, Percutaneous irreversible electroporation; MWA, Microwave ablation.

**Table 3:** VIC of the peripheral ablation area in patients with and without local tumor progression; results of the t-test for independent samples.

Ablation Technique	Mean VIC (mg/mL) in Patients with Local Tumor Progression	Mean VIC (mg/mL) in Patients without Local Tumor Progression	*p*-Value	95% Confidence Interval
IRE	1.60	0.81	0.005	0.26–1.31
MWA	1.13	0.35	0.000	0.46–1.09

VIC, Volumetric iodine concentration; IRE, Percutaneous irreversible electroporation; MWA, Microwave ablation.

**Table 4:** Comparison of the mean VIC of the peripheral ablation zone and the VIC of non-ablated liver parenchyma after IRE and MWA in each patient group: Results of the t-test for paired samples.

Local Tumor Progression	IRE		MWA	
	*p*-Value	95% Confidence Interval	*p*-Value	95% Confidence Interval
Yes	0.155	−0.24– 0.94	0.005	0.41–0.99
No	0.000	−0.90–(−0.38)	0.000	−0.99–(−0.57)

VIC, Volumetric iodine concentration; IRE, Percutaneous irreversible electroporation; MWA, Microwave ablation.

**Table 5:** Results of binary logistic regression model predicting local tumor progression.

Variable	OR (95% CI)	*p*-Value
Age	0.97 (0.88–1.06)	0.451
Tumor stage (BCLC System)	7.23 (1.67–31.22)	0.008 *
Sex: female vs. male	3.22 (0.24–43.66)	0.379
Tumor localization: segment I-IV vs. segment V-VIII	1.82 (0.29–11.47)	0.524
Ablation method: IRE vs. MWA	0.44 (0.08–2.25)	0.320

IRE, Irreversible electroporation; MWA, Microwave ablation; CI, confidence interval; BCLC System, Barcelona Clinic Liver Cancer System; *, significant *p*-value.

## Data Availability

The datasets generated during and/or analyzed during the current study can be published, as soon as the manuscript is accepted, or are available from the corresponding author on reasonable request during the review-process.
